# Guest authorship: the practice of author pooling

**Published:** 2010-03-09

**Authors:** Emmanuel Bhaskar

I wish to express my heartfelt appreciation to the editorial team of *Open Medicine* for publishing their policy on unethical authorship and for inviting readers’ feedback to improve the integrity of scientific writing.^1^ My casual discussions with friends around the globe interested in scientific publishing suggest that most people consider guest authorship to be less unethical than falsification of data. This opinion on guest authorship, along with the lack of a suitable editorial model to prevent the practice and increasing pressure to publish, means that this unethical behaviour continues to occur despite warnings issued by journal editors.

In this regard I wish to comment on the practice of author pooling, a simple method adopted by scientists to multiply their scientific publications. My information is based on offers from global scientists to include me in their author pool after they read publications related to critical appraisal for which I was the sole author. How is an author pool developed? Let us assume that 2 scientists working in different institutions join author pool A. All publications by scientist 1 will list scientist 2 as a co-author even though the latter scientist has not made a significant contribution to the work. In the same way, all publications by scientist 2 will list scientist 1 as a co-author. Each scientist’s number of publications is thereby doubled. If the author pool contains 4 scientists in 2 institutions then each scientist’s number of publications increases 4-fold.

Is there a way to halt this practice? The advancement in research databases enables articles published by individual authors to be easily identified. Suppose that the editors of *Open Medicine* decide to scrutinize the publication history of a corresponding author whose manuscript was recently published in the Journal. The analysis will reveal whether a group of individuals frequently co-author articles with the person under scrutiny. If such a group is identified, then the next step will be to ascertain the institution(s) at which the authors are based and their field of research. Journals will require a protocol for managing suspected author pooling and guest authorship. Evolving such a protocol may not be simple, but it is possible, particularly with the increasing availability of mapping tools for authors and papers in various electronic databases.

It may be helpful to invite scientists to offer their viewpoints, suggestions or experiences concerning this issue. Guest authorship should end. For this goal to be achieved we first need strategies to identify the practice. When scientists realize that they are being watched, the rate of guest authorship will definitely decrease, although the practice will not vanish.
